# Hepatitis A outbreak linked to imported frozen strawberries by sequencing, Sweden and Austria, June to September 2018

**DOI:** 10.2807/1560-7917.ES.2018.23.41.1800528

**Published:** 2018-10-11

**Authors:** Theresa Enkirch, Ronnie Eriksson, Sofia Persson, Daniela Schmid, Stephan W. Aberle, Emma Löf, Bengt Wittesjö, Birgitta Holmgren, Charlotte Johnzon, Eva X. Gustafsson, Lena M. Svensson, Lisa Labbé Sandelin, Lukas Richter, Mats Lindblad, Mia Brytting, Sabine Maritschnik, Tatjana Tallo, Therese Malm, Lena Sundqvist, Josefine Lundberg Ederth

**Affiliations:** 1 Public Health Agency of Sweden, Solna, Sweden; 2 European Programme for Public Health Microbiology Training (EUPHEM), European Centre for Disease Prevention and Control (ECDC), Stockholm, Sweden; 3 National Food Agency, Uppsala, Sweden; 4 Austrian Agency for Health and Food Safety, Vienna, Austria; 5 Center for Virology, Medical University of Vienna, Vienna, Austria; 6 European Programme for Intervention Epidemiology Training (EPIET), European Centre for Disease Prevention and Control, (ECDC), Stockholm, Sweden; 7 Department of Communicable Disease Control and Prevention, Blekinge County, Sweden; 8 Department of Communicable Disease Control and Prevention, Skåne County, Sweden; 9 The Environment and Health Administration of Stockholm Municipality, Stockholm, Sweden; 10 Department of Communicable Disease Control and Prevention, Östergötland County, Sweden; 11 Department of Communicable Disease Control and Prevention, Kalmar County, Sweden; 12 Department of Communicable Disease Control and Prevention, Gävleborg County, Sweden

**Keywords:** Hepatitis A virus, Sequence Analysis, Genotype, Foodborne Diseases, Disease Outbreaks, Sweden, Austria, Europe

## Abstract

Between June–September 2018, 20 hepatitis A cases were notified in six counties in Sweden. Combined epidemiological and microbiological investigations identified imported frozen strawberries produced in Poland as the source of the outbreak. Sequence analysis confirmed the outbreak strain IB in the strawberries with 100 % identity and the respective batch was withdrawn. Sharing the sequence information internationally led to the identification of 14 additional cases in Austria, linked to strawberries from the same producer.

Hepatitis A virus is an important cause of food-borne diseases and has been associated with several European outbreaks linked to berries [1-4]. Here, we describe an ongoing outbreak of hepatitis A virus (HAV) in Sweden and Austria and the confirmation of frozen strawberries imported from Poland as the source of infection. The aims are to highlight the importance of sequencing in outbreak investigations and, due to the long shelf-life of the food vehicle, to increase awareness and warnings towards HAV infections related to frozen strawberries in Europe.

## Outbreak identification

On 14 June 2018, the Public Health Agency of Sweden (PHAS) received a notification from the Regional Office of Communicable Disease Control and Prevention of a suspected local outbreak of HAV in County A with five cases. An epidemiological investigation was initiated by county A together with PHAS and the National Food Agency (NFA).

**Figure 1:**
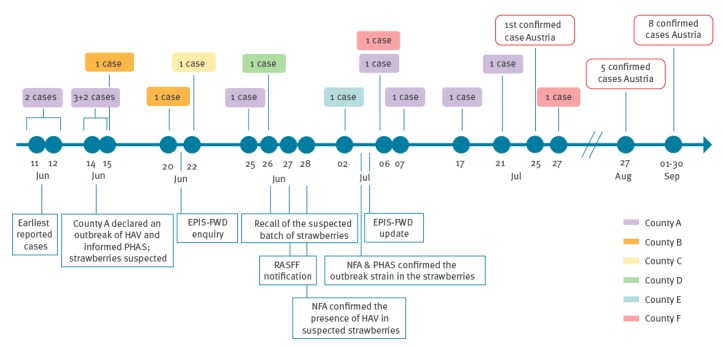
Timeline of the hepatitis A virus genotype IB outbreak in Sweden and Austria, June–September 2018 (n = 33)^a^

EPIS-FWD: Epidemic Intelligence Information System for Food- and Waterborne Diseases and Zoonoses; HAV: hepatitis A virus; NFA: National Food Agency (Sweden); PHAS: Public Health Agency of Sweden; RASFF: Rapid Alert System for Food and Feed.

^a^ Confirmed cases: Sweden 17, Austria 14; Probable cases: Sweden 2, Austria currently unknown.

Between 11 June and 27 July 2018, 20 confirmed and probable cases were reported from six counties (Figure 1). HAV genotyping of 17 of the 20 cases confirmed an identical genotype IB strain (the outbreak strain).

Cases are shown by date of notification in contrast to the epidemic curve where the date of onset (symptoms) is shown. Counties A–F refer to Swedish counties. One of the suspected cases from county A was diagnosed abroad, the exact date is unknown and therefore not listed in the timeline.

On 27 August 2018, the Austrian reference laboratory for viral hepatitis reported five cases sharing the Swedish outbreak genotype IB strain to the Ministry of Health (MOH). The Austrian Agency for Health and Food Safety (AGES) was mandated to investigate the outbreak by MOH.

## Case definition

A confirmed case was defined as a person with laboratory-confirmed hepatitis A and dates of symptoms onset after 1 May 2018 (Sweden) or 1 June 2018 (Austria) and with a sequence identical to the HAV subgenotype IB outbreak strain (MH730560, submitted to GenBank), based on an overlapping fragment of the VP1/P2A region. Probable cases were those diagnosed with hepatitis A with an epidemiological link to the strawberries of the identified producer but who were not sequence-confirmed.

## Epidemiology

**Figure 2:**
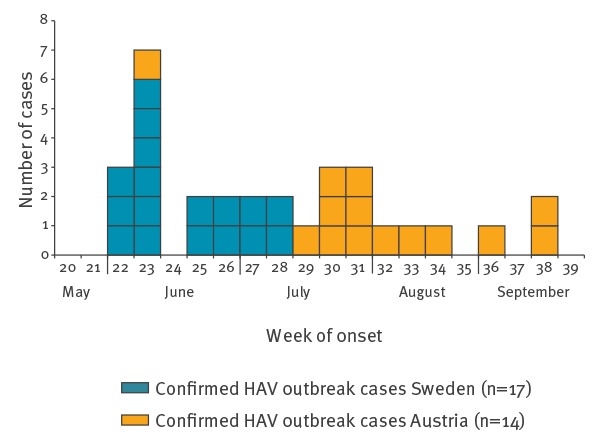
Hepatitis A virus genotype IB outbreak cases by date of onset and country of residence, Austria and Sweden, June‒September 2018 (n = 31)

As at 09 October 2018, the outbreak in Sweden comprised 20 cases, of whom 17 were confirmed cases and three were probable. Dates of symptom onset ranged from 30 May to 10 July 2018 (Figure 2). The age of the cases ranged from 9–92 years (median 41.5 years) and the majority of the cases were women (n = 13/20). All cases had domestically acquired HAV infections and came from six different counties in Southern Sweden (Figure 1). The epidemiological investigation was coordinated by PHAS and included a standard questionnaire for hepatitis A on exposures and telephone interviews, conducted by the local Regional Offices of Communicable Disease Control and Prevention. Results showed that all cases had consumed strawberries within 30 days before disease onset and at least 10 cases had consumed strawberry smoothies at two different juice bars of the same franchiser (county A). Five cases had consumed a dessert with sauce made from frozen strawberries at three different elderly homes (county A, B and E) and two cases (from county C and D) reported eating a mix of berries at a breakfast buffet at the same hotel in county A. One case had a strawberry smoothie at a restaurant (county A). The two cases from county F consumed strawberry smoothies served at a school. Environmental investigations and interviews with kitchen personnel revealed that in none of the cases had the strawberries been heated adequately before serving. Strawberries were the only food item that all the cases had in common.

HAV: hepatitis A virus.

As at 04 October 2018, a total of 36 cases of laboratory-confirmed acute hepatitis A were reported to public health authorities in Austria (since June) of which 14 fulfilled the definition of a confirmed outbreak case and four were classified as non-outbreak cases based on sequence-typing data; for the remaining 18 cases, sequence-typing data are not available and questioning regarding food exposure during their incubation period is ongoing. For the 14 confirmed outbreak cases, disease onset ranged from 8 June to 20 September (Figure 2), the median age was 21.0 years (range 5–70 years) including eight males and six females. Thirteen were residents in three neighbouring provinces in Eastern Austria. Telephone interviews using a standardised questionnaire on food exposure and purchasing behaviour were conducted with the 14 confirmed outbreak cases and 12 revealed consumption of strawberry ice cream during the incubation period. Out of these, nine remembered the restaurants where they ate the ice cream.

## Microbiological findings

In Sweden, 17 cases were confirmed by sequencing of an overlapping region (692 nt) of the HAV genome (VP1/2A; M14707 position 2683–3374), covering the 460 nt fragment recommended for typing by the global HAV network HAVNET [5]. Six cases, from counties B-F, had not visited the juice bars in county A or other franchisers connected to it (Figure 1); sequencing of those cases quickly linked them to the outbreak in county A and interviews later confirmed the cases had all consumed strawberries.

**Figure 3:**
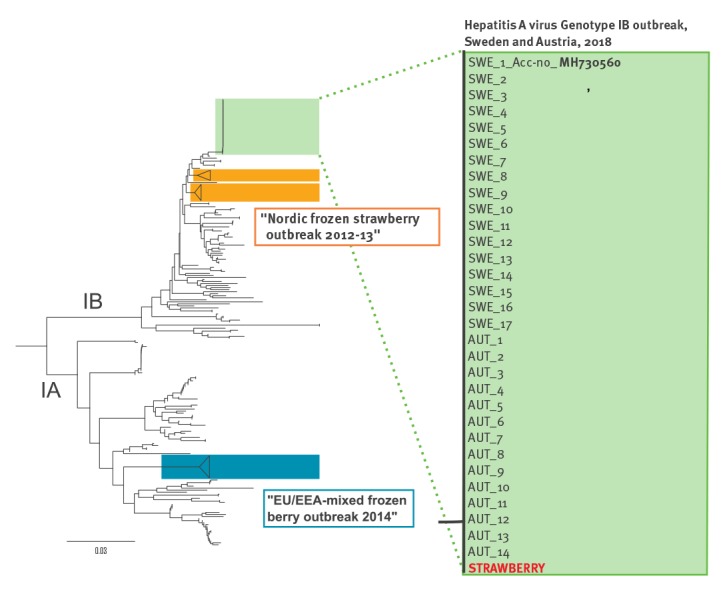
Phylogenetic tree (neighbour-joining, VP1/2A region) of hepatitis A virus genotype IB cases, Sweden and Austria, 2018

In Austria, the 14 confirmed outbreak cases were identified by sequencing the 460 nt fragment as recommended by HAVNET. Phylogeny revealed that all cases were genetically 100% identical (Figure 3).

Swedish cases of the berry-related outbreak in the Nordic countries, 2012–13 are highlighted in orange, cases of the multistate outbreak in Europe, 2014 in blue and hepatitis A virus genotype IB outbreak, Sweden, Austria, 2018 are highlighted in green.

The phylogenetic tree was constructed using VP1/2A sequences, Sweden 2013–2018 with the Neighbour-joining method in MEGA7 [22], using the Tamura Nei as evolutionary model, and a bootstrapping approach for the statistical analysis (1,000 replicates). The scale bar indicates the number of nucleotide substitutions per site.

Basic Local Alignment Search Tool (BLAST) [6] and HAVNET were used to find an indication of the geographical origin of the outbreak strain (MH730560). No similar or related sequences were identified in neither of the databases. The most closely related sequences were involved in an outbreak of HAV in travellers returning from Egypt in 2013 [7,8] and from the HAV outbreak affecting the Nordic countries in October 2012–June 2013, which was likely caused by frozen strawberries (Figure 3) [1]. However, the low sequence similarity (98%) suggests that there is no relation between these outbreaks.

## Food investigation and outbreak control

**Figure 4:**
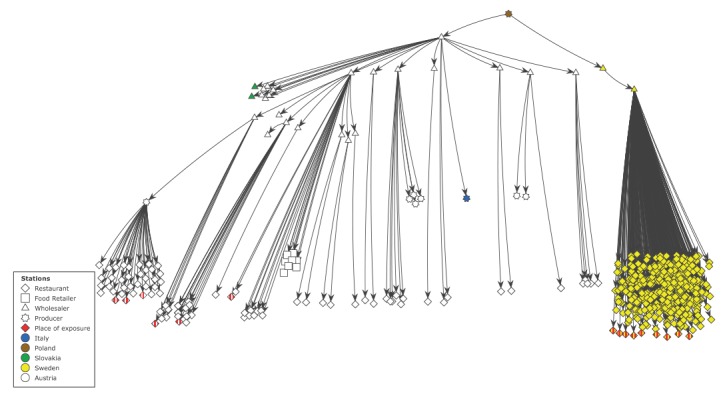
Schematic visualisation of the supply chain of frozen strawberries originating from Poland, hepatitis A virus genotype IB outbreak, Austria and Sweden, June‒September 2018

In Sweden, the Environmental Health Offices in counties A and B traced back the origins of the frozen strawberries used at the juice bars, the elderly homes, schools and at the hotel breakfast buffet (nine locations in total) (Figure 4). Food wholesaler X was identified as a common supplier of frozen strawberries to all locations. From wholesaler X, the strawberries were traced back through a trader in Sweden who had purchased frozen strawberries from producer Y, residing in Poland. Left over strawberries from the wholesaler X were found in the freezer in one of the elderly homes and were sent in to the NFA. On 26 June, wholesaler X initiated the withdrawal of the incriminated batch of frozen strawberries from the market. The batch was labelled with the best-before date 06 April 2020 (Environment and Health Administration of Stockholm Municipality, Stockholm, personal communication). In total, 1,664 packages with 5 kg strawberries each (corresponding to 8,320 kg) were withdrawn from the Swedish market.

Created with FoodChain-Lab, which is a KNIME extension providing trace back and forward analysis of suspicious food items along food supply chains in case of a foodborne disease outbreak; the open-source software was developed by the German Federal Institute for Risk Assessment (BfR) [23].

Red/white striped diamonds indicate the likely place of exposure (n = 6) of eight out of the 14 Austrian confirmed cases and the red/yellow striped diamonds the place of exposure (n = 9) of all of the 17 Swedish confirmed outbreak cases linked to the producer in Poland.

NFA did a laboratory investigation and confirmed the presence of HAV in the left over strawberries on 28 June 2018 by reverse transcription (RT) real-time polymerase chain reaction (PCR). Viral RNA was isolated and sequence analysis demonstrated the presence of the outbreak strain IB in the strawberries with 100% identity (Figure 3). The identification of the outbreak strain IB confirms the imported frozen strawberries as the common source of this outbreak.

As a consequence of the outbreak, the juice bar chain decided to stop using frozen strawberries in their smoothies and changed to pasteurised frozen pellets of strawberries instead. Staff of the juice bar chain, the elderly home and other persons who had contact with cases were tested for HAV. Other public health measures included preventive vaccination and administration of gamma globulins.

In Austria, AGES performed an extended trace back analysis focusing on the supply of the frozen strawberries used for the production of the outbreak-related strawberry ice cream. The same producer in Poland that had been identified by the Swedish authorities was identified as source of the particular frozen strawberries (Figure 4). Since mid-May 2018, only one wholesaler in Austria has purchased frozen strawberries from the producer Y in Poland—these strawberries had been further distributed to at least 13 wholesalers, two hospital food service kitchens (in Austria), two wholesalers (in Slovakia) and to one ice cream producer (in Italy). In total, about 90 restaurants and cafeterias in Austria were identified as recipients of frozen strawberries originating from producer Y in Poland (Figure 4).

Six of these restaurants and cafeterias could be linked to eight Austrian outbreak cases, through the consumption of strawberry ice cream during the incubation period. Food samples collected from stored packages of frozen strawberries originating from producer Y in Poland (best-before dates 06.03.2020 and 26.06.2020), tested negative for HAV.

The single Austrian importer of frozen strawberries from producer Y, who is expecting a frozen strawberry supply of 22 tonnes at the end of 2018, has requested evidence for a HAV-negative test result of samples from the next incoming strawberry supply. Furthermore, the Austrian importer has requested a guarantee from the purchasers that frozen strawberries are heated before consumption.

## International enquiry

On 21 June 2018, the PHAS launched an urgent enquiry in the European Centre for Disease Prevention and Control (ECDC) Epidemic Intelligence Information System for Food- and Waterborne Diseases and Zoonoses (EPIS-FWD). At that time, nine countries reported that they had not observed any HAV infections associated with the outbreak sequence (name in EPIS-FWD: ‘SW 18–09763’). The sequence was also uploaded and shared on HAVNET. On 27 June 2018, Stockholm municipal authority sent a notification to the national contact point for the European Commission’s Rapid Alert System for Food and Feed (RASFF) (Reference number 2018.1813).

On 25 July 2018, Austria reported the first case infected with the same genotype IB outbreak strain in EPIS-FWD (Figure 1).

## Discussion

In Sweden, the incidence rate for hepatitis A infections is low with an average of 0.9 cases per 100,000 population per year from 2010 to 2017 (data not shown). In 2017, it increased slightly due to the ongoing outbreak of hepatitis A disproportionally affecting men having sex with men (MSM) [9-12]. A similar incidence rate holds for Austria with an average of 0.8 cases per 100,000 population per year over the same period; in both countries, hepatitis A is a notifiable disease. In Sweden, cases fulfilling the European Union (EU) case definition for hepatitis A (IgM or HAV RNA positive, with clinical criteria of an acute hepatitis) [13] are reported to PHAS by clinicians or laboratories via an electronic system called SmiNet. Samples are send to PHAS for typing on a voluntary basis (60–70% of the notified cases). In Austria, a case fulfilling the EU case definition is reported by the clinician or diagnosing laboratory to the relevant public health authorities via a web-based, case-recording system called “Elektrisches Meldesystem” (EMS). Within the described hepatitis A outbreak since beginning of August, AGES has implemented an intensified monitoring of cases of hepatitis A registered in the EMS. This includes the prompting to transfer HAV-IgM positive serum samples of reported hepatitis A cases from the primary laboratories to the national reference laboratory (Center for Virology, Medical University of Vienna) for genotyping.

Hepatitis A virus is transmitted through faecal-oral route, via direct person-to-person contact or contaminated food and water [14]. In the current multi-country HAV outbreak with a total of 31 confirmed cases so far, imported frozen strawberries were identified as the vehicle of the outbreak agent. Several large multi-country outbreaks have been reported in the last 5 years related to contaminated berries [1-4], demonstrating that berries are a common source of contamination with HAV and suggesting that they should always be considered in HAV outbreak investigations. On 26 July 2018, almost 2 months after the initial outbreak alert in Sweden, Austria reported a case of HAV infection with a strain indistinguishable from the Swedish outbreak strain, genotype IB. As at 4 October, a total of 14 cases fulfilled the definition of a confirmed outbreak case in Austria. Due to rapid sharing of the outbreak sequence and information on food exposure in the EPIS-FWD enquiry from Sweden, strawberry ice cream was identified as the common link among the Austrian outbreak cases. Trace back investigations in Austria revealed that the respective frozen strawberries were from the same producer Y in Poland as the frozen strawberries identified as vehicle for the Swedish HAV outbreak.

There are several challenges with HAV outbreaks related to berries: first, the trace back can be difficult as berries are typically harvested by one producer, then packed by another food business operator whereby batches may then be mixed or split. In this outbreak, the country of origin of the strawberries was identified to be Poland and the wholesaler in Sweden who bought the strawberries acted quickly and withdrew the incriminated batch. Since they were only distributed to canteen kitchens or large-scale catering establishments (not to retail stores), a list of recipients was available allowing for fast withdrawal of the strawberries from the market. Nevertheless, frozen berries can be stored for a long time in the freezer or end up in multiple different dishes (desserts, smoothies, cakes, etc.), which can complicate tracing. In Sweden, the fast withdrawal together with timely public health measures such as preventive vaccination and administration of gamma globulins to contact persons at affected facilities and heating of frozen strawberries contained the outbreak. As a result, case numbers were relatively small compared with previous reported HAV outbreaks related to frozen berries (e.g. 1,803 cases in Italy, 2013/14) [4].

Although frozen strawberries were also highly suspected during the large HAV outbreak affecting the Nordic countries 2012/13, it was not possible to detect HAV in any of the strawberry samples [1]. In the current outbreak, leftovers from the suspected frozen strawberries in Sweden were sent to NFA and the presence of HAV could be confirmed by real-time PCR and Sanger sequencing, providing evidence for the source of infection. While being invaluable in tracing the source of infections, the molecular detection of viruses in food is challenging [15,16]. Reverse transcription (RT) real-time PCR is the standard method for monitoring viral contamination in food [17], but strawberries often contain inhibitors which cause an inefficient amplification of the RNA target sequence [18]. Moreover, the viral load in berries is often low and the viral particles have to be concentrated before performing RT real-time PCR. Many concentration methods are inefficient and may cause co-purification of RT and/or PCR inhibitors present in the food sample [19]. These factors in combination can lead to an underestimation of the viral load as described for other enteric viruses [20]; false negative results are therefore common.

Another point to consider during HAV outbreak investigations is the long incubation period of hepatitis A (up to 6 weeks). After such a long time, it can be difficult to recall food consumption and collect food leftovers. Furthermore, the time from the onset of symptoms to a primary diagnosis and the sequence information can take several weeks.

This HAV outbreak linked to imported frozen strawberries gives reason to re-evaluate the national recommendations for the handling of frozen berries in Sweden. HAV remains infectious during frozen storage and washing with water before consumption has limited effects in removing HAV from berries and herbs [21]. During the large HAV outbreak due to frozen berries in the Nordic countries in 2012/13, the NFA in Sweden recommended to heat frozen strawberries before consumption to inactivate enteric viruses. After the outbreak, this recommendation was dismissed for strawberries. However, the NFA recommends consumers and food business operators to boil frozen, imported raspberries due to the risk of norovirus. The current outbreak demonstrates that boiling recommendations could be considered for frozen strawberries, or all frozen berries as in Denmark, Finland and Norway [1]. This recommendation has been also given in the current hepatitis A outbreak by the Austrian public health authorities.

## Conclusion

In conclusion, the combined epidemiological and microbiological investigations and food trace back analyses identified frozen strawberries as the vehicle of the two-country HAV outbreak. In Sweden, the findings led the wholesaler to voluntarily withdraw this product from the Swedish market. While there were no further reported cases in Sweden since 27 July 2018, the latest outbreak case in Austria occurred in calendar week 34. Based on findings of the trace back analyses identifying supply of the particular frozen strawberries to Slovakia and Italy, occurrence of further cases in other European countries is possible. Increasing awareness will help to accelerate a fast public health response.

This report highlights the importance of fast collaborations between different agencies, and the crucial role of sharing sequencing data of HAV samples between European countries. Without the sequence information, it would have been difficult to allocate single cases in county B-F and cases in Austria to the same outbreak.
